# Supplementary material for: Molecular and morphological survey of Lamiaceae species in converted landscapes in Sumatra
Source: PLoS One. 2022 Dec 15;17(12):e0277749. doi: 10.1371/journal.pone.0277749 (PMC9754244; doi:10.1371/journal.pone.0277749)
Supplement: S1 Table — (DOCX) [file pone.0277749.s001.docx]

**S1 Table. Sequence information and characteristics of the individual markers *matk*, *rbcL* and ITS.**

| **Parameter** | ***matK*** | ***rbcL*** | **ITS** |
| --- | --- | --- | --- |
| Number of samples used for amplification and sequencing | 89 | 89 | 89 |
| Number of obtained sequences | 73 | 72 | 41 |
| Variable Sites (proportion) (%) | 39% | 18% | 52% |
| Parsimony informative sites (proportion) (%) | 23% | 10% | 34% |
| CG content mean (range) (%) | 35.9% | 45.1% | 62% |
| Length of the alignment, bp | 755 bp | 589 bp | 838 bp |
