# Supplementary material for: Molecular and morphological survey of Lamiaceae species in converted landscapes in Sumatra
Source: PLoS One. 2022 Dec 15;17(12):e0277749. doi: 10.1371/journal.pone.0277749 (PMC9754244; doi:10.1371/journal.pone.0277749)
Supplement: S2 Table — (DOCX) [file pone.0277749.s002.docx]

**S2 Table. The availability of reference sequences for each of the species used in this study.**

| **Species** | ***matK*: BOLD** | ***matK*: NCBI** | ***rbcL*: BOLD** | ***rbcL*: NCBI** | **ITS: NCBI** |
| --- | --- | --- | --- | --- | --- |
| *Callicarpa candicans* | All mined from GenBank | YES | NO | NO | YES |
| *Callicarpa pentandra* | All mined from GenBank | YES | All mined from GenBank | YES | YES |
| *Clerodendrum infortunatum* | All mined from GenBank * | YES | All mined from GenBank. *Also, BOLD sequences | YES | YES |
| *Clerodendrum cf. haematolasium* | N/A | NO | N/A | NO | NO |
| *Clerodendrum deflexum* | NO * | NO * | NO * | NO * | NO |
| *Clerodendrum laevifolium* | NO * | YES | NO * | YES | YES |
| *Clerodendrum myrmecophilum* | N/A | NO | N/A | NO | NO |
| *Clerodendrum ridleyi* | N/A | NO | N/A | NO | NO |
| *Gomphostemma cf. parviflorum* | N/A | NO | N/A | NO | NO |
| *Hyptis capitata* | One mined from GenBank * | YES | NO * | NO | YES |
| *Peronema canescens* | NO * | NO | NO * | NO | YES ** |
| *Sphenodesme triflora var. triflora* | N/A | NO | N/A | NO | NO |
| *Teijsmanniodendron coriaceum* | Samples were ‘early BOLD release’ | YES | NO * | YES | NO |
| *Teijsmanniodendron pteropodum* | N/A | NO | N/A | NO | YES |
| *Vitex gamosepala* | NO | NO | NO | NO | NO |
| *Vitex pinnata* | NO | YES | All mined from GenBank | YES | YES |
| *Vitex quinata* | All mined from GenBank | YES | All mined from GenBank | YES | YES |
| *Vitex vestita* | NO * | NO | NO * | NO | YES |

N/A: No results available. YES: Direct match available. NO: No direct match available, but results shown. *Sequences from our specimens uploaded to NCBI by our research group. **ITS1 only.
