# Supplementary material for: Molecular and morphological survey of Lamiaceae species in converted landscapes in Sumatra
Source: PLoS One. 2022 Dec 15;17(12):e0277749. doi: 10.1371/journal.pone.0277749 (PMC9754244; doi:10.1371/journal.pone.0277749)
Supplement: S3 Table — (DOCX) [file pone.0277749.s003.docx]

**S3 Table. Conservation status of each species used in this study.**

| **Species** | **Conservation Status** |
| --- | --- |
| *Callicarpa candicans* (Burm.f.) Hochr. | LC |
| *Callicarpa pentandra* Roxb. | LC |
| *Clerodendrum cf. haematolasium* Hallier f. | DD |
| *Clerodendrum deflexum* Wall. | DD |
| *Clerodendrum laevifolium* Blume | LC |
| *Clerodendrum myrmecophilum* Ridl. | DD |
| *Clerodendrum ridleyi* King & Gamble | DD |
| *Clerodendrum villosum* Blume | DD |
| *Gomphostemma cf. Parviflorum* Wall. ex Benth. | DD |
| *Hyptis capitata* Jacq. | DD |
| *Peronema canescens* Jack | LC |
| *Teijsmanniodendron coriaceum* (C.B.Clarke) Kosterm. | LC |
| *Teijsmanniodendron pteropodum* (Miq.) Bakh. | LC |
| *Vitex gamosepala* Griff. | LC |
| *Vitex pinnata* L. | LC |
| *Vitex quinata* (Lour.) F.N.Williams | LC |
| *Vitex vestita* Wall. ex Walp. | LC |
| *Sphenodesme triflora var. triflora* Wight | DD |

LC= Least concern. DD= Data deficient
