# Supplementary figures and images for: Molecular and morphological survey of Lamiaceae species in converted landscapes in Sumatra
Source: PLoS One. 2022 Dec 15;17(12):e0277749. doi: 10.1371/journal.pone.0277749 (PMC9754244; doi:10.1371/journal.pone.0277749)

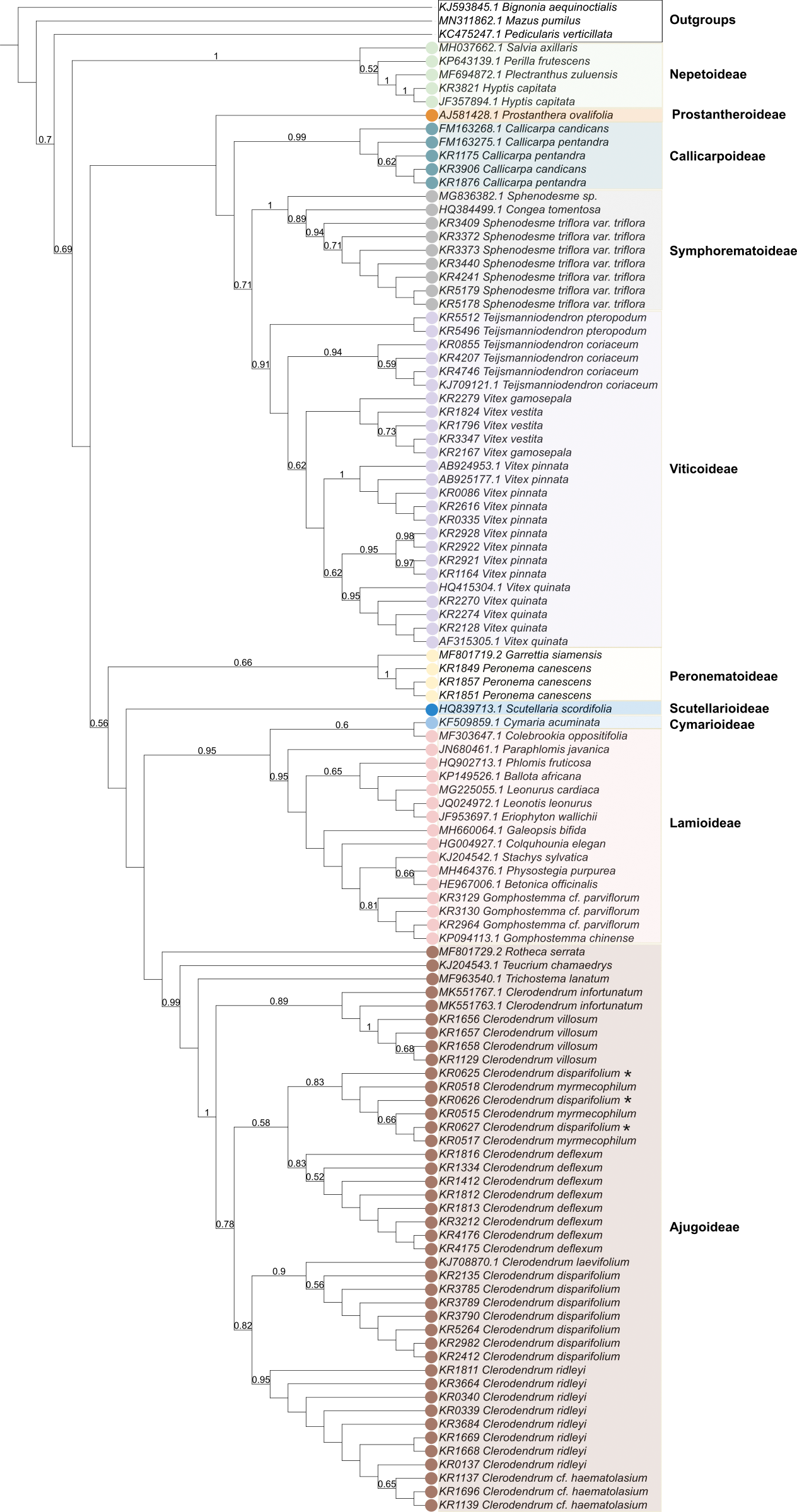

Supplement: S1 Fig — The tips of the tree labels display the species IDs. The subfamilies of the Lamiaceae family are highlighted in different colours. * Highlights juvenile specimens. (TIF) [file pone.0277749.s004.tif]

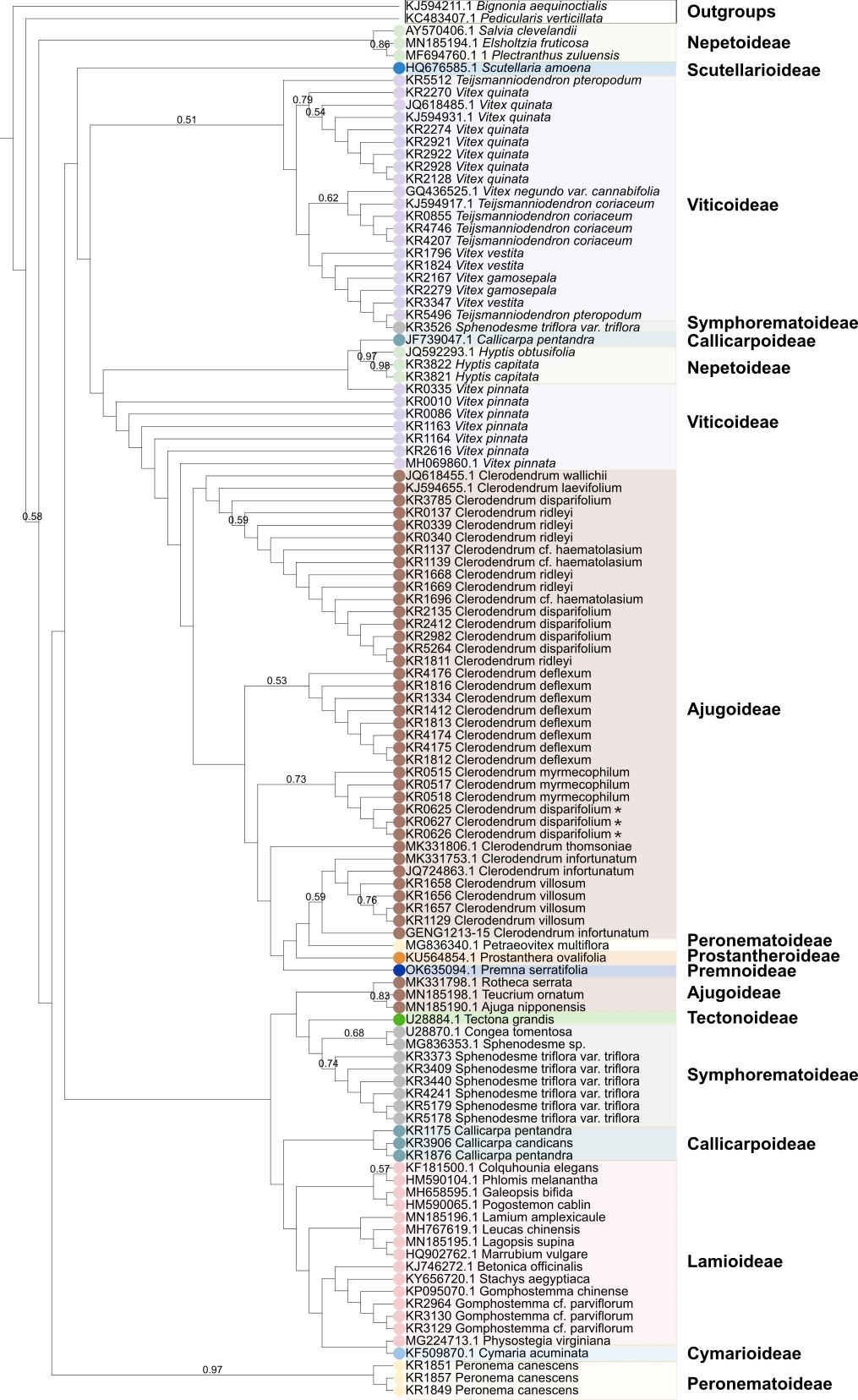

Supplement: S2 Fig — The tips of the tree labels display the species IDs. The subfamilies of the Lamiaceae family are highlighted in different colours. * Highlights juvenile specimens. (TIF) [file pone.0277749.s005.tif]

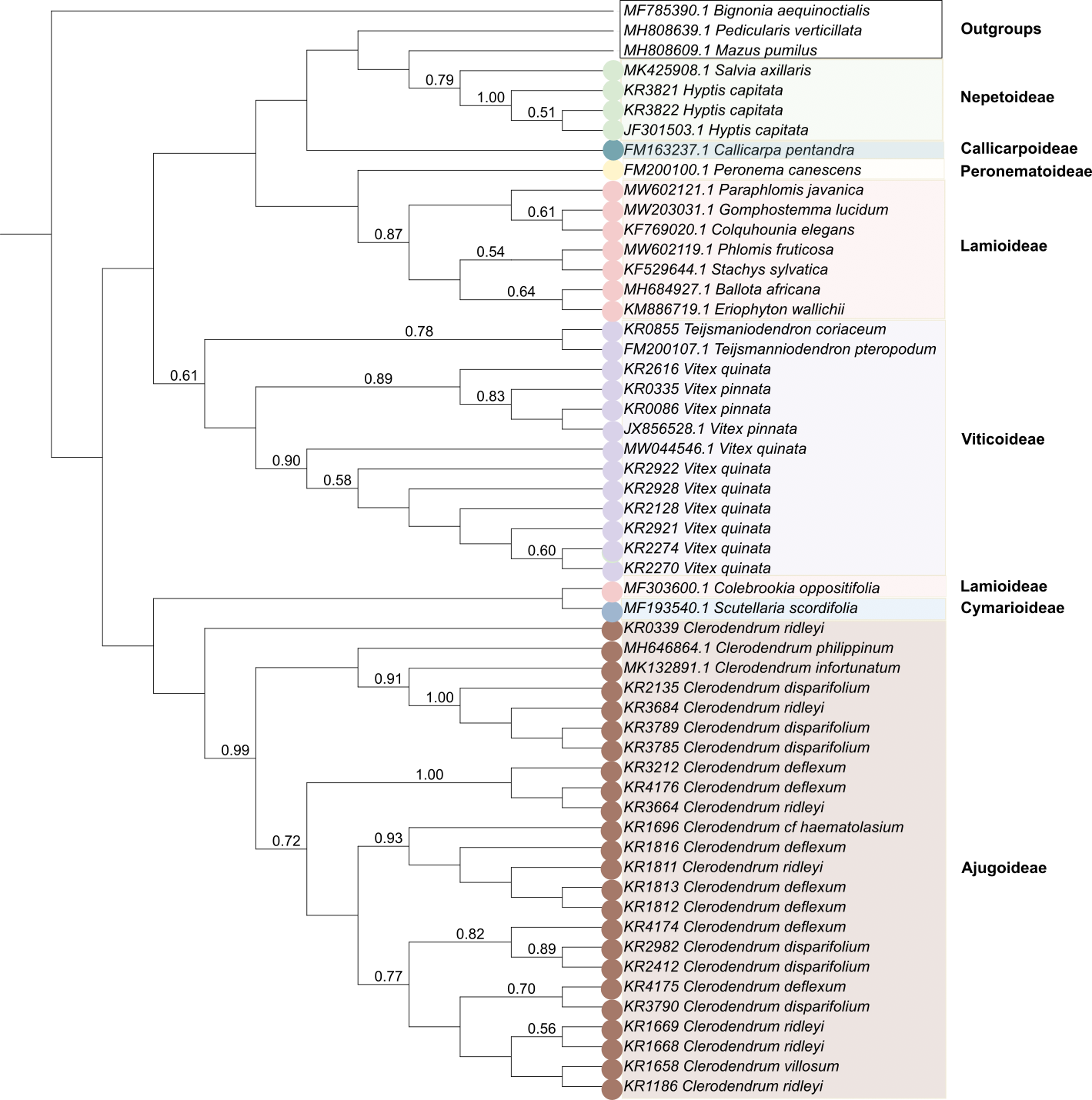

Supplement: S3 Fig — The tips of the tree labels display the species IDs. The subfamilies of the Lamiaceae family are highlighted in different colours. (TIF) [file pone.0277749.s006.tif]

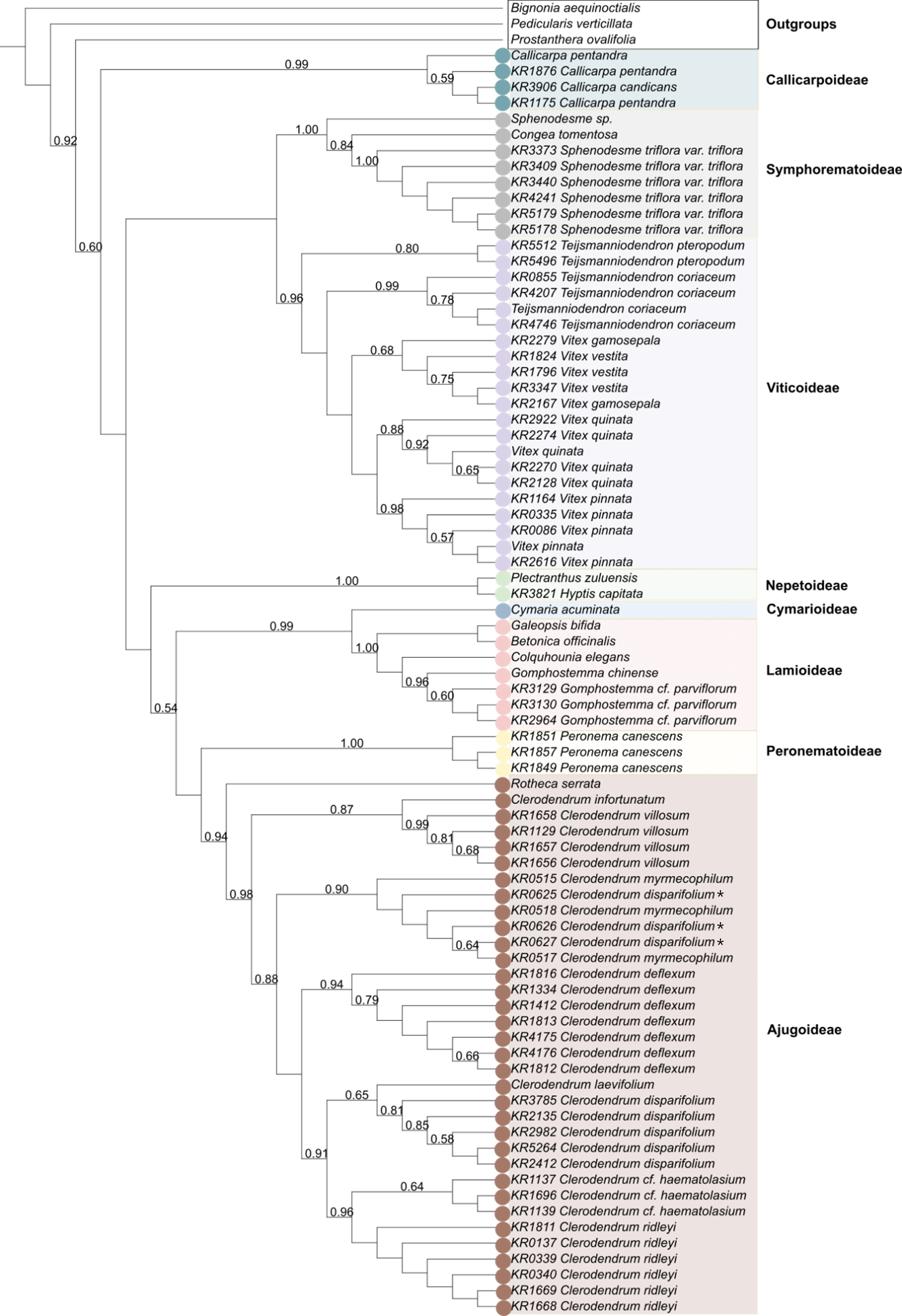

Supplement: S4 Fig — The tips of the tree labels display the species IDs. The subfamilies of the Lamiaceae family are colour highlighted. * Highlights juvenile specimens. (TIF) [file pone.0277749.s007.tif]
