# Supplementary material for: Molecular and morphological survey of Lamiaceae species in converted landscapes in Sumatra
Source: PLoS One. 2022 Dec 15;17(12):e0277749. doi: 10.1371/journal.pone.0277749 (PMC9754244; doi:10.1371/journal.pone.0277749)
Supplement: Attachment — Submitted filename: PLos 1 MS sumatra.docx [file pone.0277749.s008.docx]

The manuscript entitled “**Molecular and morphological survey of Lamiaceae species in converted landscapes in Sumatra**" comprises informative and valuable work. The study is useful in view of harnessing the potential of the species of Lamiaceae in **Sumatra and other region of the world. It is also important to resolve taxonomic ambiguities within the member of this family** and help to establish phylogenetic relationships among the related species/genera. Manuscript is well written and presented nicely. Data analysis is sufficient to draw conclusion and interpretation of the data is good. Based on observations during reviewing this manuscript, I recommend the manuscripts for minor revision to enhance the scope of the MS among readers. To improve the quality and scope of the paper, the following minor comments and suggestions are required to be addressed by the authors

- Abstract should be clearer and appropriate Rewrite it should have one or two concluding line and recommendation based on this study.
- Reduce the size of introduction it’s too lengthy.
- How the leaf sample was collected from the field. Zip lock bag with slica gel was used to transport the samples from field to lab or anything else please describe in the relevant section
- Replace mix with master mix in method.
- Conclusion part also require more precise summary of the results and expected outcomes of the study.
- Overall, manuscript is well written pure scienfic and provides novel information and hence recommended for acceptance with minor revision.
