# Supplementary material for: Molecular and morphological survey of Lamiaceae species in converted landscapes in Sumatra
Source: PLoS One. 2022 Dec 15;17(12):e0277749. doi: 10.1371/journal.pone.0277749 (PMC9754244; doi:10.1371/journal.pone.0277749)
Supplement: Attachment — Submitted filename: Response letter to reviewers.docx [file pone.0277749.s009.docx]

# **Response to Reviewers**

We are grateful for the valuable contribution of the editor and reviewers. We addressed all raised points, and point-by-point response are added below:

The manuscript entitled “**Molecular and morphological survey of Lamiaceae species in converted landscapes in Sumatra**" comprises informative and valuable work. The study is useful in view of harnessing the potential of the species of Lamiaceae in **Sumatra and other region of the world. It is also important to resolve taxonomic ambiguities within the member of this family** and help to establish phylogenetic relationships among the related species/genera. Manuscript is well written and presented nicely. Data analysis is sufficient to draw conclusion and interpretation of the data is good. Based on observations during reviewing this manuscript, I recommend the manuscripts for minor revision to enhance the scope of the MS among readers. To improve the quality and scope of the paper, the following minor comments and suggestions are required to be addressed by the authors

Author’s answer: Dear reviewer, thank you very much for your constructive review, we appreciate your time. We are positive that your suggestions helped us to improve the quality of our manuscript since we have addressed all the points raised. The main goal of this study was to provide a proof of concept by emphasizing the applicability of barcoding regions for biodiversity inventories in tropical areas.

- Abstract should be clearer and appropriate Rewrite it should have one or two concluding line and recommendation based on this study.

Author’s answer: We restructured the abstract and included further conclusion information as recommended.

- Reduce the size of introduction it’s too lengthy.

Author’s answer: We agree and have shortened the introduction.

- How the leaf sample was collected from the field. Zip lock bag with slica gel was used to transport the samples from field to lab or anything else please describe in the relevant section

Author’s answer: We added the further details. Indeed, the samples were transferred in sealable bags containing silica gel.

- Replace mix with master mix in method.

Author’s answer: Corrected.

- Conclusion part also require more precise summary of the results and expected outcomes of the study.

Author’s answer: We added further information regarding the relevance of the study, summary of our findings and expected outcomes as requested.

- Overall, manuscript is well written pure scienfic and provides novel information and hence recommended for acceptance with minor revision.

Author’s answer: We appreciate your positive feedback. We also decided to improve the figures, which look clearer than the previous version.

Further comments:

Regarding data accessibility, we will include the accession numbers during the proof revision of the manuscript. The sequences have been submitted to NCBI and accession numbers are included in this version of the manuscript.

The manuscript was formatted according to the requirements of the journal. The licenses for this study were included in the methodology as required. No human samples or data was collected.

Yet again, we appreciate the dedicated time of the reviewers and editor for the improvement of this paper and acknowledge their contributions to the quality of our research article.

Sincerely,

Dr. Carina Moura
